# *Sharing our story* individualized and triadic nurse meetings support couples adjustment to living with deep brain stimulation for Parkinson’s disease

**DOI:** 10.1080/17482631.2020.1748361

**Published:** 2020-04-09

**Authors:** Anita Haahr, Annelise Norlyk, Elisabeth O. C. Hall, Kirsten Elisabeth Hansen, Karen Østergaard, Marit Kirkevold

**Affiliations:** a Research Centre for Health and Welfare Technology, Programme for Rehabilitation, VIA University College, Aarhus, Denmark; b Public Health, Nursing, Aarhus University, Aarhus, Denmark; c Faculty of Health Sciences and Nursing, University of the Faroe Islands, Tórshavn, Faroe Islands; d Department of Oncology, Aarhus University Hospital, Aarhus, Denmark; e Department of Neurology, Aarhus University Hospital, Aarhus, Denmark; f Department of Nursing Science, Faculty of Medicine, University of Oslo, Oslo, Norway; g Institute of Nursing and Health Promotion, Oslo Metropolitan University, Oslo, Norway

**Keywords:** Parkinson’s disease, nursing intervention, nursing, deep brain stimulation, advanced treatment, patient and spouses perspective, adjustment

## Abstract

Treatment with deep brain stimulation for Parkinson’s disease, leads to a rapid improvement in mobility, which may challenge patients and spouses when adjusting to everyday life. An intervention, developed to support the adjustment to everyday life with DBS, demonstrated that individualized meetings with a specialized nurse was experienced as important and fruitful by both patient and spouses. **Purpose:** The aim was to gain a deeper understanding of how the meetings contributed to the adjustment process. **Method:** 38 audio-recorded meetings and six written summaries from eight couples participating in the intervention, were analyzed in a hermeneutic process. **Results:** The analysis revealed four themes: A relational triad of co-creating personal knowing. Sharing and listening in an atmosphere of trust and openness. Unveiling the couple’s everyday life, coping strategies and expectations. Supporting adjustment through knowing their personal story. **Conclusion:** The triadic dynamics in the meetings were quite particular. The main focus was the patients’ and spouses’ stories, individually and as a couple. The DBS nurse pursues solutions based on professional and specialized knowledge of Parkinson’s disease and the couple’s everyday life. Thus, the intervention meetings offered tailored, individualized and specialized care in supporting adjustment to DBS for PD both individually and as couples.

## Background

Parkinson’s disease (PD) is a chronic progressive neurodegenerative disease (Kalia & Lang, 2017). Main motor symptoms are resting tremor, rigidity, bradykinesia and postural instability (Hughes et al., 1992; Kalia & Lang, 2017). Initially PD is medically treated with dopamine agonists including levodopa, but when this becomes insufficient, deep brain stimulation (DBS) might be an option (*Medtronic*, 2017; Weaver et al., 2009). Intermittent loss of medication effect leads to an unpredictable motor function, ultimately leaving patients in an unstable everyday life (Caap-Ahlgren & Dehlin, 2002; Haahr et al., 2011; Wressle et al., 2007). This does not only affect the quality of life (QoL) of patients (Benge et al., 2016; Prakash et al., 2016; Van Uem et al., 2016), spouses too are significantly affected by their partners’ illness (Birgersson & Edberg, 2004; Haahr et al., 2013).

Advanced treatment of PD using DBS has been an option worldwide for more than two decades for patients living with advanced PD (*Medtronic*, 2017). DBS improves motor symptoms of PD and QoL and reduces motor fluctuations (Deuschl et al., 2006; Volkmann et al., 2009; Weaver et al., 2009). It is considered a safe treatment when based on careful selection of candidates (Krüger et al., 2016; De Rosa et al., 2016). The main reason for accepting DBS treatment is intolerable motor complications and the perception that DBS is the last chance of improvement, as medical opportunities have become insufficient (Haahr et al., 2010; Hamberg & Hariz, 2014; Mathers et al., 2016).

A distinct feature of successful DBS treatment is the rapid improvement in mobility, leaving patients with a feeling of a miracle (Haahr et al., 2010; Mathers et al., 2016). However, patients are still living with PD. The few studies addressing the patients’ experiences reveal that psychosocial aspects are challenging; patients may experience identity problems, difficulties adjusting to changes in physical ability, difficulties adhering to new treatment strategies and, in some cases, new symptoms. Patients also express a continuous need for professional support (Haahr et al., 2018; Hariz & Hamberg, 2014; Hariz et al., 2016). Thus, DBS challenges everyday life with PD; it frequently contributes to new “miraculous” opportunities, but also challenges both patients and spouses. Thereby, it underlines a need for tailored preparation both before and after DBS surgery (Eatough & Shaw, 2017; Haahr et al., 2010; Knoop et al., 2017)

### A psychosocial intervention to support adjustment to DBS for PD

Based on a previous study identifying that patients undergo an adjustment process the first year after DBS, (Haahr et al., 2010) we developed a psychosocial intervention adhering to British Medical Research Council (MRC) guidelines (Craig et al., 2008). The aim was to support patients and their spouses in adjusting to everyday life with DBS for PD. The intervention lasted six months and addressed DBS from a patient and spouse perspective (Haahr et al., 2018). Inspired by Zoffmann’s Person-Centred Communication and Reflection Model (Zoffmann et al., 2008), the intervention meetings intended to facilitate adjusting to everyday life changes, in particular by integrating life-oriented and disease-oriented perspectives of everyday life with PD during the process of adjusting, for both patients and spouses. Besides the targeted, individual meetings with the DBS nurse, the intervention included journaling and a leaflet about the adjustment process (Haahr et al., 2018). The content of the intervention is illustrated in Table I.

**Table I.** The intervention content and structure (Haahr et al., 2018, p. 178)

Meeting number	Focus area	Content of meeting	Structure; physical or phone meeting	Approx. time for meeting
First meeting	***Everyday life at present and expectations for DBS***	● daily life at present ● coping with PD ● expectations	Meeting between patient, spouse and DBS nurse Lasting approx. one hour	2–4 weeks before DBS
Second meeting	***Changes in body, illness and everyday life.***	● time immediately after DBS ● present challenges ● managing changes ● managing life and disease	Meeting between patient, spouse and DBS nurse Lasting approx. one hour	4–6 weeks after DBS
Telephone meeting	***Follow-up phone meeting***	● evaluating goals ● adjusting goals ● managing problems at present ● making arrangement for next meeting	Telephone meeting Lasting approx. 15–30 minutes	6–8 weeks after DBS
Third meeting	***Taking responsibility for DBS now and onwards*** ***Living with DBS in the future***	● focus on own resources and wishes for the future ● setting realistic goals ● looking back: now and then	Meeting between patient, spouse and DBS nurse Lasting approx. one hour	3 months after DBS
Telephone meeting	***Managing changes***	● identifying present questions ● supporting the ability to make choices and decisions	Telephone meeting Lasting approx. 15–30 minutes	4 months after DBS
Fourth meeting	***Summarizing and evaluating***	● everyday life with DBS at present ● everyday life in the long term ● evaluating participation in the intervention	Meeting between patient, spouse and DBS nurse	6 months after DBS

NOTE: The Creative Commons license does not apply to this content. Use of the material in any format is prohibited without written permission from the publisher, Wolters Kluwer Health, Inc. Please contact permissions@lww.com for further information.

Each intervention encounter had a specific focus guided by the adjustment process (Haahr et al., 2010). Nurses who participated in the study received training in how to perform the intervention and had worksheets with questions to guide and facilitate each meeting (Haahr et al., 2018). An example of topics and questions guiding the second meeting (first meeting *after* DBS) is illustrated in Table II.

**Table II.** An example of questions guiding the nurses at the second meeting (4–6 weeks after DBS)

Second meeting (first meeting after DBS): Changes in the body, illness and everyday life
*Content and topics:*
Changes in body and illness after DBS Changes in treatment strategy Challenges that patients and spouses experience after DBS Addressing expectations, wishes and goals for DBS
*Questions that may be addressed by nurses:*
How do you experience your everyday life with DBS treatment right now? How does DBS affect your everyday life? How do you manage changes in your everyday life, because of the DBS treatment? Have you engaged in any new activities since the surgery? Have you continued with your previous activities? Do you think about the DBS stimulation as part of your treatment? How do you experience the connection between stimulation, medication and symptoms? How do you experience your body? Does it react differently? If yes, what does that mean to you? What are your expectations to everyday life with PD and DBS for the next year?

To evaluate the intervention, a feasibility study was first performed. The feasibility study focused on analysing the meaningfulness and content of the intervention from the patients’ and relatives’ perspective (Haahr et al., 2018). A significant finding was that the individualized meetings with the DBS nurse were emphasized as the most valued part of the intervention and seemed to help facilitate change processes. In order to gain a deeper understanding of how the meetings contributed to the adjustment process, the present study explored the meetings in further detail.

## Method

The paper reports on a qualitative evaluation, focusing on potential benefits and harms of the intervention, reflecting phase three of the MRC framework (Craig et al., 2008; Ludvigsen et al., 2013). Understanding in more detail why these meetings were so appreciated by the patients and spouses may contribute with crucial knowledge for developing future sound and useful interventions.

### Participants

Eight couples going through DBS for PD participated in the meetings as part of the intervention. Two female and six male patients with PD, ranging from 56 to 68 years of age, and two male and six female spouses. Disease duration of the participating patients ranged from 8 to 17 years at time of DBS. Two patients and two spouses were employed at time of DBS. Three experienced DBS nurses from two hospital settings performed the intervention.

### Ethics

Patients and spouses received oral and written information about the study. They were assured that participation was voluntary and that they could withdraw from the study at any time without explanation. All participants signed a letter of consent. The study protocol was presented for the regional health research ethics committee, who had no objection to the study.

### Data collection

The data consisted of audio-recorded conversations between the nurse and the couples. In one of the nurse-couple meetings, data were not recorded as participants were most comfortable without a recorder. Instead, résumés of their meetings were made. Otherwise, all intervention meetings between the DBS nurse and the couple were audio-recorded. In five couples, two telephone meetings with each couple were also recorded. In all, the data material consisted of twenty-eight audio-recorded meetings lasting from 30 to 90 minutes audio, ten recorded telephone meetings and written summaries of six meetings.

### Data analyses

The analysis followed Gadamer’s (1991) interpretation of hermeneutics as a circular and spiral form of moving between the parts and the whole of a text, and between presuppositions, interpretations and applications of the target phenomenon. To understand why the intervention meetings were so appreciated, we aimed to interpret data from the meetings with an open mind to create a new understanding. Gadamer states that valid interpretation has an effect only if a text is understood “in a different way as the occasion requires” (Gadamer & Silverman, 1991, p. 309). Therefore we interpreted the text, in this case data from the meetings, in the context of aiming to understand the characteristics and meaning of the meetings, and secondly how the meetings affected the experiences and expectations of the participants.

We formulated the following analytical questions: What characterized the meetings? What was the content of the meetings? What characterized the relation between the participants and which knowledge about patients’ and spouses’ ways of managing changes after DBS were created in the meetings? Guided by these questions, we looked at data for each participating couple presented in a descriptive text and through listening to the recorded meetings. To identify similarities and discrepancies, we analysed the data across couples and across a timespan for each couple. The analysis was thus performed in a hermeneutic process of reading the descriptive texts, reading data from across participants and listening to the recordings. We continuously addressed and discussed the merging themes in an ongoing dialogue among authors, until a comprehensive and deeper understanding of both parts and whole emerged.

## Results

The analysis revealed interacting characteristics of the meetings that each had a significant impact on the meetings and was considered valuable to support adjustment to DBS for PD. The characteristics are described in four themes: *A relational triad of co-creating personal knowing. Sharing and listening in an atmosphere of trust and openness. Unveiling the couple’s everyday life, coping strategies and expectations. Supporting adjustment through knowing their personal story.*

### A relational triad of co-creating personal knowing

One significant characteristic was that the meetings constituted a triad of consequential relations where the multiple relations were quite striking. Thus, this was not only a matter of the nurse meeting the couple. The meetings revealed a relational triad constituted by sincere and fundamental encounters between the nurse and patient, the nurse and spouse, among the couple, and the nurse and the couple. The meetings thus proved to be a setting were the nurse could relate to each of the participants individually, giving the opportunity to explore the experience of living with PD after DBS from both the patient’s and the spouse’s perspective. This opportunity provided insights of what it is like living with PD after DBS and the challenges experienced by patients as well as the experiences of being a spouse whose partner is going through DBS for PD. Thus, what was quite novel was that the nurse could focus exclusively on the spouse. Also, by meeting the patient and spouse together, experiences and thoughts were shared, not only between the couple and nurse, but more notable, among the couple. Thus, the triadic meeting provided insights into the couples’ adjustment and coping with illness on several levels. As examples, the meetings revealed how the couple made everyday agreements about how the healthy partner should or should not help the partner with PD, how they managed issues related to PD or how they arranged holidays or daytrips to make sure PD would not be a hindrance. The meetings also described how couples analysed difficult situations together and how a few of the couples turned to alternative medicine to deal with symptoms related to PD. This knowledge provided valuable insights as to how the couples coped with the illness. One DBS nurse reflected on this with one couple:

I have learned more about your way of living your life. That you do not focus too much on illness during your everyday life. I have got a sense of you focusing on the alternative that I would not have known otherwise.

The couples reported that they valued the togetherness. They had learned new things about each other and their ways of managing life with PD both before and after DBS and as such the relational triad proved to open up for a dialogue among the couple. One participant said: *“It has made us think differently in the process. We had hopes and expectations, but our goals for the DBS treatment were not explicit for us”* (wife, couple 3). Thus, the triad revealed that the meetings initiated a process of adjusting for both patients and spouses.

### Sharing and listening in an atmosphere of trust and openness

An open and trustful relation among the parties was established already in the first meeting. The meetings reflected that the patients willingly shared their experiences of living with PD as well as their private and personal views. Patients gave their first-hand experiences of living with PD, such as: *“The worst part is the ups and downs—the constant change between ON and OFF. It is annoying!”* (husband, couple 5). The nurse used information like this to support patients in adjusting to DBS by helping the patients to formulate goals and expectations. The DBS nurse also highlighted agreements and goals made; and evaluated if they had been achieved. If not, the nurse analysed, with the patient, reasons for not achieving the desired goals and suggested new goals, with the couple, based on their needs, resources and wishes. One nurse summarized the first meeting after DBS with one couple like this: *”I think you are doing fine. Your OFF episodes are not so dominant anymore. Being active is a good strategy for you, and it is ok for you to be tired”*. Then, the nurse explained that being tired could be a reaction to the physical and emotional challenges that the patient had been going through, thus using professional knowledge in an individualized manner.

The nurses also attended specifically to the spouses. An important feature of the triadic relation was the unique focus on the spouses’ everyday life, their thoughts, fears and wishes when living with a partner with PD. Thus, the relation between nurse and spouses in the meeting was experienced markedly different from the usual nurse-spouse relation in caring encounters. One spouse expressed the experience like this: “*The fact that one nurse is beside you all the way and that you know each other, makes you feel safe and secure”* (wife, couple 7). And a husband stated:

*After going through an operation like that, it is nice to come and be able to talk to people who know about it. It makes you feel more secure. Once you are out of the system, you are on your own* (husband, couple 4).

Evolving around everyday life as *a couple* living with PD, the nurse acknowledged PD as having significant impact on family life and them as a couple. Some couples explicitly mentioned how the meetings helped them address new mutual knowledge, giving them opportunity to share thoughts and be open to each other in ways they did not usually. At the meetings, they disclosed thoughts, feelings and coping strategies for each other:

*… the funny thing is that we get to talk about stuff. Some things are too hard to talk about, and then indirectly, we get to see things anyway. That is the way it is. And it is a good thing. I did not know that she had been hallucinating. She never told me. But now I know* (husband, couple 1).

The meetings also opened for discussions of aspects of life that were not initially addressed as part of the intervention: *“The meetings have been good, because sitting there, talking about things, we sometimes discussed other issues that we came to think of”* (wife, couple 4). These issues were often related to other things, such as worrying about serious illness in the family or worrying about the health of grandchildren; they impacted on the couples’ adjustment and signalled that everyday life revolved around more than Parkinson’s.

### Unveiling the couples’ everyday life, coping strategies and expectations

Even though the meetings sometimes solely focused on physical aspects of the disease, an important feature was that they still centred on individual experiences. Everyday life got a prominent place in the meetings. Several participants remarked on this matter; it helped them feel more involved in the treatment and adjustment. It seemed that the meetings had a similar structure across participants. The nurse began by asking how the couples were doing, mostly focusing on the patient first. By doing this, the nurse often got an initial reply relating to PD symptoms or the state of the disease, like *“my tremor has gone”* or *“I am still struggling with my balance”*. The focus on everyday life with PD was perceived as valuable; it contributed to trust, understanding and adjustment to the disease changes. One spouse stated:

*It has been good for us to be part of this, having these discussions that are broader …. after each session we have a different perception of what we can and cannot do. Because we have discussed it, and you (the nurse) know something about these things and we can relate to that. We might not do as you suggest. But we can reflect on it and we get to talk about different things more than we would have otherwise* (husband, couple 1).

An example of how the couples shared experiences was their structuring of everyday life when one partner was working, how they then divided chores between them and their motivation for doing so. The couples described how they made room for exercise, how they planned going out for dinner, how they managed OFF periods or how they might be drawn to alternative medicine, just to feel that they were trying something themselves too. They told about sharing life stories with children and grandchildren and they described fears and worries about the future. Generally, they revealed diverse and unique coping strategies. One participant said:

*I have learned to look after myself a bit more. I feel better then. I don’t feel comfortable being with too many people at one time. It freaks me out. So, I have to take notice of that* (wife, couple 1).

What seemed to be general was a focus on the present. Almost all participants shared the reluctance to plan or think too much ahead. When asked, one participant described what he thought of the future: *“I don´t. I just take each day as it comes. I plan maybe a week or a month ahead. No more”* (husband, couple 5). His wife agreed, though she aimed to anticipate a bit further ahead. Despite their focus on the present, it was evident that they had hopes and dreams for the DBS treatment; this was an integral part of deciding to go through DBS. One spouse stated:

*We have to remember that he has Parkinson’s. When he has several good days running, we start planning a whole lot. Suddenly one day I had to stop myself. In my mind I kept thinking “when he gets well” … regardless that I know it is a chronic illness. … it was then I started to look at it differently. Getting there was not easy* (wife, couple 7).

Thus, during the meetings, the nurse not only offered care and trust, the couples were offered alternative ways to understand their situation; and they were supported in balancing hope and challenges.

Additionally, the meetings revealed that also the spouses go through an adjustment after their partners’ DBS. Giving voice to and listening to the spouses supported them in verbalizing *their* expectations, coping strategies and resources. This was exemplified in several meetings. One spouse described how they both unknowingly had adjusted to the challenges associated with PD. Now, she felt challenged by not having to control her husband’s everyday life but also by the new possibilities that so instantly had become a part of their everyday life:

*“Before, if we were going somewhere, I often went on my own. Actually, often it was best if I just said “I’m going”. Without asking him. Now he may want to come along and I have to remember that. It is nice, but I have to get used to thinking that he might want to come too.”* (wife, couple 3).

### Supporting adjustment through knowing their personal story

The fourth characteristic of the meetings was that together, the nurse, patient and spouse created personal knowing of everyday life before and after DBS. The nurse used insights from the diary and previous meetings to learn more about each person’s story with PD, and these data became the basis from which interpersonal knowing emerged. The joint experience, as a couple, was recalled as important and significant. Often patient and spouse touched on sensitive subjects. In such cases, the nurses were offering the couple to withdraw from the subject, but it never happened. On some occasions, the nurse turned off the recorder, only to turn it back on as the couple wished to continue. Usually, the couple continued talking about the subject arguing that *“it is good and necessary to talk about it”*. Thus, when confronted with more sensitive aspects of life, one of the spouses stated:

*“ … well of course I worry. Even though he might be irritating at times, I would like to keep him … [laughs] … . We have been together forever … Luckily, I am strong [the nurse agrees] so I hope I can keep on”* (wife, couple 6).

In general, the nurses integrated this personal knowing to give information targeted at the couple; helping them to act differently or to see new opportunities, and supporting them in getting a better understanding of the ongoing changes in body, illness and symptoms post DBS. The nurse’s holistic approach was essential when setting goals and verbalizing difficult hopes and expectations. Participants described how they were caught up in a tension between hopes and dreams and try to be realistic. Neither patients nor spouses knew what to expect. However, the meetings reflected an atmosphere where it was legitimate to verbalize all kinds of expectations. The nurse respectfully acknowledged this tension by offering a way of achieving goals or modifying expectations. One patient e.g., wished to significantly reduce his medication; he felt bothered by side effects. The nurse responded by giving him an estimate of the reduction of medication. Using the couple’s individual experiences, the nurse formulated a realistic goal, honestly what the couple could expect. The adjustment then, was not only physical but also highly emotional.

Thus, this theme unfolds essential aspects of the meetings as relational and characterized by a holistic approach where the interrelatedness of the themes becomes evident. The relational triad, co-creation of knowledge, the trustful atmosphere and the revealing of individual resources are all aspects of creating personalized, individualized meetings where each of the couples tells their unique story.

## Discussion

The aim of this paper was to reach an in-depth understanding of the significance of the series of post DBS intervention meetings between DBS nurses, PD patients and their spouses. The idea was to explore how integrating the patient’s and spouse’s narratives may support their adjustment to DBS. Describing characteristics, content and relations inherent in the meetings, we found that the essential characteristic of the meetings was a positive and trustful atmosphere through which the relational triad (nurse, individual, couple) could be co-creating further personal knowing about the illness, the treatment and life itself. Trust, sharing and openness among the parties created a supportive arena where individual resources, coping strategies and expectations unfolded and goals and individual agreements were decided upon.

As previously documented, the intervention was inspired by the person-centred communication and reflection model, developed by Zoffmann and colleagues (Zoffmann et al., 2008). This model entails four steps in person-centred communication and reflection: accomplishing focused communication, integrating life-oriented and disease-oriented perspectives, moving from non-situational to situational reflection and finally reaching deeper levels of situational reflection characterized by mutual or independent patient reflection (Zoffmann et al., 2008, p. 673). The findings of this study seem to reflect Zoffman’s focused communication, as the participants emphasized that they experienced the meetings as evolving in an atmosphere of trust and openness, based on mutual relations with focus on promoting growth and adjustment to a future life with DBS through individual goalsetting. The meetings were integrating both life-oriented and disease-oriented perspectives. We found that a precondition for the triad relation to thrive in these meetings was that nurse, patients and couples experienced a connection, which also others have described as necessary for engaging in a relationship that promotes growth and comfort (McMahon & Christopher, 2011; Miner-Williams, 2007). Hereby acknowledging how emotionally and physically demanding the process of adjusting to DBS for PD is. The significance of this demand can be further strengthened with reference to Hellqvist (2015) who identified the need for support from a specialist PD nurse in four categories: professional competence, nursing practice, continuity of contact and emotional support. These categories were all integrated parts of an overarching category described as *“Competent, professional practice, tailored for the individual”* (Hellqvist & Berterö, 2015, p. 88). Essential attributes of these four categories are described as; focus on the individual, care-orientation, daily life, familiarity and trust, personal relation and guidance to both patient and spouses. Thus, Hellquist’s categories have great similarity to the relationship among nurse, patient and spouses in our study. Furthermore, they seem to be somewhat comparable to Zoffmanns person-centred care (Zoffmann et al., 2008). Galvin and Todres (2013) offers a conceptualization of lifeworld-led care that has a focus on well-being in a positive sense that may provide a direction for care (Galvin & Todres, 2013, p. 6). Their approach may support and underline the need for interventions and meetings like in this study, focusing on a relational understanding of the specific situation. Galvin and Todres argue that an “*embodied relational understanding refers to a way of knowing that is holistically contextual”* (Galvin & Todres, 2013, p. 148). An important aspect of this relationship may be understood as related to “a complex use of self” as described by Todres et al. (2014). This means that the nurse needs to understand the vulnerability and what the patient is going through, being someone that the patient can trust (Todres et al., 2014), which calls for an ability to actively listen to the patient and spouse.

Also Hariz and Hamberg (2014) identified participants’ need for continuous contact with a DBS nurse, however, mainly related to getting assistance with adjusting and tuning the DBS stimulation (Hariz & Hamberg, 2014). A qualitatively different aspect was revealed in our study, where the findings reflected that adjustment to DBS involves an emotional process. Several of the participants in our study expressed a need for the meetings after the intervention ended (Haahr et al., 2018), underlining a need for continuous contact with a DBS nurse to support patients and spouses in living well with DBS for PD. Thus, regardless of the motor improvement following DBS, there is a need for a psychosocial follow-up. Consequently, our findings highlight the need for a long-lasting close relation between nurse and couple, beyond the timeframe of our intervention of six months.

### Involvement of the individual and the couple as a key feature

Involving patients and spouses in the meetings, focusing on their story and their experience of everyday life with PD, was a fundamental idea in this study (Haahr et al., 2018; Zoffmann et al., 2008). In this case, involvement meant mutual and personalized engagement of the patient and spouse, aiming at giving the patient and spouse a direction for goalsetting based on their individual needs and wishes, and acknowledging that patients may feel lack of control and insecurity during the process of adjusting to DBS (Haahr et al., 2010, 2018). Finding it hard to express what to expect post DBS, studies also stress patients’ and spouses’ need for involvement in the process of receiving DBS, both pre- and post-surgery (Eatough & Shaw, 2017; Gilbert et al., 2017; Haahr et al., 2018; Knoop et al., 2017; Weernink et al., 2016). Involvement thus is considered important to ensure that patients and spouses know what to expect, and to align their expectations (Haahr et al., 2010). In this study, the predetermined focus for the intervention meetings guided the discussions of certain issues. However, the participants felt that there was time and space to tell their individual story. The meeting atmosphere was open and trustworthy. Through telling their stories, participants not only felt involved by the nurse, they felt involved by each other. This is a significant finding of this study. The specific setting for these meetings, involving both patients and spouses, offered a unique involvement of the patient and spouse as a couple. The arrangement allowed the couple to reach deeper levels of understanding their own needs and wishes, leading to, what Zoffmann et al. (2008) describe as situational reflection. In other words, in discussing and addressing current challenges of adjusting to DBS for PD, the participants had the possibility to reach a deep level of focused communication (Zoffmann et al., 2008). Thus, the meetings not only supported the individualized process that patients go through and their need for tailored care (Haahr et al., 2010; Weernink et al., 2016), the meetings also enhanced what they go through together and what is involving them as a couple. Having a family member present may support the individual in living with illness (Årestedt et al., 2018). Knowing that spouses to persons living with Parkinson’s disease may be at risk of filling double roles as both spouse and caregiver, it is imperative to invite spouses to healthcare encounters. Martin (2016) found that given the opportunity to work together in coping with Parkinson’s disease, bring couples closer together (Martin, 2016). Thus, involving both patients and spouses is an important feature of the intervention. According to Buetow et al. (2016), the matter of involvement is closely linked to person-centred care, based on the professional and personal involvement of all parties in each specific case (Buetow et al., 2016). Their, as well as our findings in this study, reflect involvement at different levels, especially related to knowing the personal stories of those involved. Sometimes the nurse takes control and guides the patients and spouses, other times the nurse pushes the couple to come forward. The findings thus reflect the significance of involvement in the meetings; and it is only successful when involving time and space to patients *and* spouses, both individually and as a couple. We would argue, that involvement is first genuine when the nurse is trusted, open and listening, acting upon expressed needs and wishes and known to guide towards realistic and desirable decisions. Thus, the nurse’s involvement, in meetings like those of our study, has strong connotations to a “caring conversation” as described by Fredriksson and Eriksson (2003), where the mutual involvement may be understood as an ethical encounter (Fredriksson & Eriksson, 2003).

### Listening and reflection

The relational triad, as one significant finding in this study, was characterized by respect among all three parties. Challenges, concerns, and meanings were discussed, planned or just listened to. This openheartedness indicates that the structured and set meetings provided time and space for the nurse, patient and spouse to meet and focus on life with PD and DBS. The findings reflect that through listening and having a specific focus on lifeworld perspectives, a different understanding of the couples’ everyday life, and their coping strategies such as turning to alternative medicine, was created among participants in the meeting. The ability to listen, may be seen as an essential aspect of the person-centred reflection model as this is a precondition for moving from non-situational to situational reflection (Zoffmann et al., 2008). When listening genuinely the nurse actively enters the world of the patient (Hooper, 2013), giving specific or individual advice or suggestions to the couple. This is based on knowing the couple, and it also reflects that the intervention has provided them with a suitable frame to do this. As the couple and the nurse were given a certain focus for the meetings, this served as a platform from which the meetings could evolve. Even though it differed depending on how well prepared the participants were, they had a mutual understanding of what the meeting would be about.

As the dialogue of this study was a triad, there may be certain challenges in getting all parties to engage in both compassionate listening and sharing. However, the findings highlighted the important relational work of the specialist nurse, and they shed light on the valuable professional competencies of a nurse working with PD patients and their spouses (Hellqvist & Berterö, 2015).

### Creating their personal narrative to support adjustment

The relational triad had the unique feature of involving both patient and spouse thus integrating the spouse’s specific need for involvement (Haahr et al., 2013). The meetings integrated spouses differently than in routine meetings within the healthcare system, and this was highly appreciated by both spouses and patients (Haahr et al., 2018, p. 2). Through the meetings, the nurse, patient and spouse shared hopes, expectations and wishes for the future, or, in some cases, lack of trust in the future. It was obvious that the couples had anticipated the nature of DBS and life after DBS and that they were restructuring their life routines to a new reality. In doing so, they were trying to maintain continuity and create new choices. When the nurse acted upon these efforts by giving suggestions and supporting each couple in managing the changes of everyday life after DBS, it lead to mutual reflections as described by Zoffmann and colleagues (Zoffmann et al., 2008). According to Benner and Wrubel (1989), being with the patient and spouse, understanding what the disease is interrupting, (in this case, how DBS for PD is affecting everyday life), are essential issues when supporting patients in coping with changes in illness. Likewise based on Benner and Wrubel’s notions, knowing the person, their everyday life with PD and their expectations for DBS are essential in supporting the adjustment and coping with life changes following DBS for PD (Benner & Wrubel, 1989). Several studies report experiences of changes in self and everyday life following DBS, and the authors emphasize the importance of a holistic, individual and personal focus to support the process of adjusting to DBS (Eatough & Shaw, 2017; Gilbert et al., 2017; Kraemer, 2013).

Our findings reflect that the nurses, as well as the structure and content of the meetings are meaningful and significant in bringing forth the persons, and what it is to be a patient, a spouse and a couple with PD. Together the findings are offering an individual as well as a couple’s perspective on living with PD post DBS. Involving the triad within the meetings makes all parties acquainted with resources, expectations and coping strategies. Facilitating features of such meetings are the close connection between the patient’s and the spouse’s stories, and not least the focus on the couple’s story as well as each couple member’s recognition of the process. We argue that the spousal adjustment is an important finding, as DBS has shown to be a life changing event for the entire family. Both pre- and post-operative preparation of both patient and spouse are repeatedly suggested (Eatough & Shaw, 2017; Haahr et al., 2013, 2010; Knoop et al., 2017; Shahmoon & Jahanshahi, 2017).

Finally, when addressing the significance of involving patients and spouses, it is important to address the notion of context and competency. We have argued that the context of the meetings, the structure and content, are suitable for enhancing adjustment after DBS. We have also argued that mutual involvement and a focus on the couple’s story characterize the meetings. The relation between nurse, patient and spouse is fundamental. The set timeframe, context and content provide a platform from which this relation develops, involves and evolves.

This intervention thus has proven to be feasible and meaningful in the context of a pilot study (Haahr et al., 2018), where the meetings were experienced as the most valued part of the intervention. This study unfolds the importance of the relations created between nurse, patient and spouse in the meetings and supports a holistic approach to adjusting to DBS, integrating disease-oriented and life-oriented perspectives. Next step in the MRC approach (Craig et al., 2008) would be to test and implement this intervention in a wider context and maybe adjusting the intervention to local circumstances.

## Strength and limitations

The study is based on data from eight couples from two different hospitals. The participants represent variation and reflect the population of patients going through DBS with regard to gender, age and disease duration. The participants in this study were between 56 years and 68 years of age, and had been living with the disease between 8 and 17 years. This is comparable to other studies. deSouza et al. (2015) reports the timing of DBS for PD in UK from 1997–2012 to be static over a 15 year period. They describe a mean age of 60 years, ranging from 32–80 years. Mean disease duration to be 11.5 years, ranging from 1–34 years (deSouza et al., 2015). Thus, we expect that the experiences of the participants and the concerns voiced may be relevant for other couples with PD receiving DBS. However, this study does not cover the whole range of experiences among persons being treated for DBS due to PD. Furthermore, the study had an exclusive focus on the experiences of couples, leaving out the perspectives of patients living on their own and other family members of persons with severe PD. This may be considered a limitation of the study. Another matter is that the expert DBS nurse is decisive for facilitating the meetings which might cause diversity in how the intervention is performed. These aspects must be taken into consideration when applying an intervention similar to this one.

## Conclusion

One significant finding is the genuine relationship between the nurse and the couple. Their triadic dynamics are quite particular, supporting patients’ and spouses’ adjustment to DBS for PD. The pre-established focus for the meetings gave the triad mutual foci. The patients’ and spouses’ stories, individually and as a couple, were the content and main focus of the meetings; and these issues were guided with the help of professional ethical engagement combined with a mutual connectedness and involvement among all three parties. By pursuing patients’ and spouses’ meeting statements, in-depth knowledge is generated of how PD is affecting everyday life of the individual both pre- and post- DBS. Thereby, opportunities and space are created for the couples to express concerns and challenges; they are given opportunities to reflect on own and each other’s actions in managing these problems. In the meetings, the DBS nurses take leadership in engaging in the couple’s life story; they are pursuing solutions based on professional and specialized knowledge of PD and each couple’s unique life, health and illness situation. Thus, intervention meetings of this kind are recommended; they offer tailored, individualized and specialized care in supporting patients’ and spouses’ adjustment to DBS for PD both individually and as couples.
